# Bridging the gap between recommendation and reality: Improving dietary adherence of heart failure populations a cross-sectional study in Ethiopia

**DOI:** 10.1371/journal.pone.0311663

**Published:** 2024-10-10

**Authors:** Takla Tamir, Jemberu Nigussie, Migbaru Endawoke

**Affiliations:** Department of Nursing, Dilla University College of Health Science and Medicine, Addis Ababa, Dilla, Ethiopia; Mulungushi University, ZAMBIA

## Abstract

**Background:**

Heart failure (HF), a complex condition arising from impaired ventricular function, necessitates strict adherence to dietary recommendations for optimal patient management. However, information regarding adherence and its influencing factors remains limited.

**Aim:**

This study aimed to assess dietary recommendation adherence and its associated factors among HF patients at Southern Ethiopia public hospitals.

**Methods and results:**

A cross-sectional study involving 521 participants employed systematic random sampling. Data collection utilized pre-tested, interviewer-administered questionnaires and medical chart reviews. Data were entered and analyzed using Epi Data 3.1 and SPSS 20.0 software. Descriptive statistics were performed. Variables with p-values < 0.25 in binary logistic regression were included in multivariable logistic regression analyses. Statistical significance was set at p < 0.05 with a 95% confidence interval. Results are presented in text, tables, and figures.

With a 97.4% response rate, adherence ranged from 20.3% (vegetables and fruits) to 60.3% (fat-free diet). Only 8.1% achieved good adherence across all parameters, with overall adherence at 33.4% (95% CI: 29–37). Multivariable analysis revealed that patients aged 41–60 years (AOR: 1.7), with a history of admission (AOR: 2.5), free from comorbidities (AOR: 0.58), and possessing a favorable attitude (AOR: 0.45) had statistically significant associations with good adherence.

**Conclusion:**

Dietary adherence among HF patients remains a challenge. Healthcare providers, particularly those in chronic follow-up settings, should prioritize improving patient attitudes towards proper dietary practices. Tailored education programs targeting younger patients and those free from comorbidities should be implemented. Continuous monitoring, evaluation, and staff recognition for effective client counseling are crucial.

## Introduction

Heart failure is a chronic condition that weakens the heart’s ability to pump blood [1–5]. It can affect either the left, right, or both ventricles of the heart, and can be acute or chronic [6]. Symptoms include shortness of breath, difficulty breathing when lying down, and fatigue [2,6]. Despite advances in treatment, heart failure remains a burden on healthcare systems [7,8] due to frequent hospitalizations and reduced quality of life for patients [9,10]. While medication can slow the progression of heart failure, self-care measures like following a recommended diet are also important for managing the condition [7,11]. Although adherence to self-care can be inconsistent [3,12], studies have shown that participating in long-term self-care activities can improve outcomes for heart failure patients [9,10,13,14] and reduce the burden of the disease on individuals, families, and society [15,16].

Dietary changes are crucial for managing heart failure, especially reducing sodium and fat intake, and managing fluids [17,18]. A low-sodium diet (around 2–3 grams per day) is proven to improve symptoms and overall health for heart failure patients [19,20]. Dieticians can recommend specific sodium and fluid intake goals based on individual needs [16,20–24] Sodium intakes above and below the recommended range is associated with increased cardiovascular risk [25,26]. While some fat is necessary, a relatively low intake, particularly omega-3s from fish, may help prevent or slow heart failure progression failure [7]. On the other hand, refined carbohydrates and sugary drinks increase the risk of heart failure, while whole grains offer some protection [27].

In addition to dietary changes, guidelines recommend lifestyle modifications to manage heart failure [28]. The American Heart Association (AHA) emphasizes a healthcare system approach that encourages healthy behaviors like a balanced diet rich in fruits, vegetables, and whole grains. This aligns with recommendations for moderate alcohol intake, limited red meat and processed foods, and avoiding sugary drinks grains [17,29]. International guidelines also advise restricting salt and fluids for heart failure patients [30,31].

Heart failure is a global pandemic affecting at least 26 million people worldwide [32] and is increasing in prevalence with an impact on healthcare costs [15,16,33] due to its greatest reason of hospitalization [34]. Morbidity and mortality rates due to heart failure are increasing globally [18]. The incidence of HF increases with age, approximately from 20 per 1000 individuals with age 65 to 69 years to more than 80 per 1000 individuals aged 85 years [35].

Heart failure (HF) is a major public health problem in Ethiopia, affecting a significant number of people, primarily middle-aged adults [36] (Tenaadam & Demissei, 2018). Rheumatic heart disease is the most common cause of HF in the country [36,37]. A study of 496 HF patients revealed an in-hospital mortality rate of 24.4% [37]. Another study reported a 90-day all-cause mortality rate of 52.3% among 283 HF patients [38]. Health-related quality of life (HRQoL) is poor among Ethiopian HF patients, with 54% reporting low scores on the Minnesota Living with Heart Failure Questionnaire[39].

Heart disease accounted for 32.2% of the burden of cardiovascular disease [40]. Being non-compliant with at least one of the non-pharmacological recommendations had a higher risk of mortality or HF readmission [41]. The role of nutrition in the prevention and improvement of HF prognosis is recognized and noncompliance to sodium and fluid-restricted diet accounts for 30%–44% of readmission on HF patients [42,43].

The 2015 United States (US) Dietary Guidelines Advisory Committee recently concluded that a healthy dietary pattern is higher in vegetables, fruits, whole grains, low-fat or nonfat dairy, seafood, legumes, and nuts; moderate in alcohol; lower in red and processed meat; and low in sugar-sweetened foods and drinks and refined grains [17,29]. Several factors, Age, co-morbidity, and knowledge, influence adherence of heart failure patients to their recommended diets [44]. Understanding these factors is crucial for effective management plan and intervention implementation.

Previous research on adherence to self-care practices among heart failure (HF) patients in Ethiopia has been limited [45,46], Existing studies have focused on general self-care behaviours in a single institution with small sample sizes, making them less specific and hindering the development of targeted interventions. To address this gap, this study aimed to assess adherence to dietary recommendations and its associated factors among HF patients in Southern Ethiopia. The findings from this study can inform policymakers and healthcare providers in designing interventions to improve dietary adherence and ultimately, patient outcomes.

## Materials and methods

### Study design, area and period

An institutional-based cross-sectional study was carried out in Gedeo zone and Sidama Region, Southern Ethiopia public hospitals. The study was conducted at 7 selected public hospitals from April to June 2022.

### Population

All adult HF patients attending all public hospitals in Gedeo Zone and Sidama Region were the source population and those source populations attending selected public hospitals of Gedeo Zone and Sidama Region during the study period were the study population.

#### Eligibility criteria

Participants must meet criteria: (1. Be an adult aged 18 years or older, 2. Have a confirmed diagnosis of heart failure by a healthcare professional and 3. Have had at least one documented follow-up visit at least a week prior to study enrolment) were included in the study where as participants were excluded from the study if they have had a recent follow-up visit within the data collection period at other included hospitals or if they have severe or uncontrolled comorbidities that would significantly impact their ability to participate in the study or accurately provide data.

### Sample size determination and sampling procedure

The sample size for each objective was calculated by using the StatCalc function of Epi-Info version 7 software. The maximum sample size was 486 from the second objective and after adding a 10% non-response rate, the total sample size became 486+49 = 535.

To determine sample size allocation, we conducted a pre-recruitment survey at each participating hospital. This survey, along with a review of the past 3 months’ chronic follow-up data, allowed us to assess patient flow (daily, weekly, and monthly). Based on this data, a proportional sample size was allocated to each hospital.

For participant selection, we employed systematic random sampling. We calculated the sampling interval (k) as N/n (population size divided by desired sample size), which in this case was k = 2. Using a random lottery method, we selected the first participant. Subsequently, every other patient following the selected participant was included in the study.

### Data collection tool, operational definitions, and procedures

Data were collected using a structured, interviewer-administered questionnaire developed by adapting and combining validated instruments from previous research [18,35,47–49]. The questionnaire addressed five key areas: sociodemographic information, clinical profile, dietary adherence, knowledge about HF, and attitude towards recommended diets.

Dietary adherence was assessed using the validated "Revised Heart Failure Compliance Scale" which focuses on adherence to low sodium, fat-free, fluid restriction, legumes, and fruits/vegetables diets [18,35,47,49] This instrument demonstrated good reliability and validity [18,35,41,47,49–51].

Adherence to dietary recommendations was assessed using a five-item (a low sodium diet, fat free diet, fluid restriction, legumes, and fruits and vegetables) questionnaire with a five-point Likert scale **(**always = 4, mostly = 3, half of the time = 2, seldom = 1, never = 0**)**. For each item participants were categorized as "adherent” if they reported following a recommendation "always or mostly and for the overall as "adherent" if they adhered at least three of the five recommendations [18,35,47].

Knowledge of heart failure was assessed using the "Japanese Heart Failure Knowledge Scale" [18,48]. Cronbach’s alpha for internal consistency is 0.79. Knowledge about HF was assessed using a questionnaire with 11 yes/no/"don’t know" options. Scores above 75% were categorized as "good knowledge," [45,46].

Attitude towards recommended diets was assessed using six -item questionnaire with a five-point Likert scale (ranging from "very pleasant" to "very unpleasant"). Attitude towards recommended diets was assessed using six -item questionnaire with a five-point Likert scale (ranging from "very pleasant" to "very unpleasant"). Participants scoring above the mean score were classified as having a "favorable attitude." [45].

Interviews were conducted privately following participants’ healthcare appointments, adhering to COVID-19 prevention measures. Medical charts were reviewed to gather additional clinical data. Data collectors were nurses with BSc or higher degrees and supervisors were MSc nurses. All underwent training by the principal investigator on the study aims, questionnaire content, participant selection, data collection procedures, and ethical considerations. Data collection was performed by the trained nurses with close supervision by the principal investigator and supervisors throughout the process.

### Ethics statement

Letter of ethical clearance was obtained from institutional review board of Dilla University; college of medicine and health sciences (duirb004/21-11). Approval was obtained from the participating hospitals. Informed, voluntary, written and signed consent was obtained from each respondent. Confidentiality of the information was kept throughout the study that data were analyzed anonymously. This study was done in accordance with declaration of Helsinki.

### Data quality control

To ensure data accuracy and reliability, we implemented rigorous quality control measures throughout the research process. The questionnaire, originally developed in English, was translated to Amharic and back-translated to verify consistency. Experts in nutrition and internal medicine reviewed it for clarity and content validity. A pilot test on 5% of the sample size ensured the instrument’s effectiveness. Data collectors and supervisors received comprehensive training to minimize errors during collection. The principal investigator reviewed a portion of the collected data daily to identify and address any immediate issues. Finally, data entry staff verified the completeness, accuracy, clarity, and consistency of questionnaires before entering them into EpiData software. Double data entry was performed for verification, and data cleaning procedures addressed outliers, missing values, and inconsistencies before analysis.

### Method of data processing and analysis

After checked, coded, entered into Epi data, validated, and compared to the original, data were exported to the Statistical Package for Social Science [SPSS] Version-20 software for analysis. Descriptive analysis was done to compute proportions and summary measures. Texts, tables, and figures were used to present the processed information.

In bivariate analysis, crude odds ratio with 95% CI, was estimated to see the crude association between each independent variable with the dependent variable. All variables with P < 0.25 at a 95% confidence level during the bivariate analysis were selected as a candidate for the multivariable analysis to control all possible confounders. The multi-co-linearity test was carried out to see the linear correlation among independent variables by using standard error. Standard error >2 was considered suggestive of the existence of multi-co-linearity and no multi-co-linearity was detected. Hosmer -Lemeshow goodness- of- fit was done to check model fitness. The Omnibus test was significant (p-value = 0.000) and Hosmer- Lemeshow’s test was insignificant (p-value > 0.35) which shows the model has fitted.

An adjusted odds ratio with 95% CI was estimated during multivariate analysis to identify factors associated with adherence to the dietary recommendation. Independent variables at the level of statistical significance P < 0.05 were reported as factors having a statistically significant association with adherence to the dietary recommendation. We used the STROBE cross sectional reporting guidelines described in S1 Table [52]. Information about the dataset used for the analysis of this study is also described in S2 Table.

## Result

### Socio-demographic characteristics

A total of 521 participants were successfully interviewed, representing a 97.4% response rate. Participant ages ranged from 18 to 90 years old, with a median age of 45 (IQR = 25). Males comprised the majority of participants (52.6%), and approximately 28% reported being unable to read or write (Table 1).

**Table 1:** Socio-demographic characteristics of participants for adherence to dietary recommendation and associated factors among adult HF patients at public hospitals of, Southern Ethiopia 2022 (n = 521).

Variables		Frequency (n)	Percent (%)
**Age group (in years)**	18–40	207	39.7
	40–60	205	39.3
	>60	109	20.9
**Sex**	Male	274	52.6
	Female	247	47.4
**Educational level**	Unable to read and write	145	27.8
	Able to read and write	141	27.1
	Elementary	128	24.6
	Secondary &above	107	20.5
**Marital status**	Unmarried	87	16.7
	Married	362	69.5
	Divorced and Widowed	72	13.8
**Residence**	Urban	296	56.8
	Rural	225	43.2
**Occupation**	Gov,t employee	54	10.4
	Private employee	123	23.6
	Farmer	128	24.6
	house wife	115	22.1
	Merchant	51	9.8
	Other (student, orphans and tiered)	50	9.6

### Clinical characteristics

Over half (58.2%) of participants reported being diagnosed with heart failure for more than 4 years, indicating a long-standing health concern. Additionally, approximately 40.3% had a history of hospitalization, suggesting potential disease severity in some cases. Nearly 43% of participants lived with at least one additional chronic condition, with hypertension being the most prevalent, followed by diabetes mellitus (Table 2).

**Table 2:** Clinical characteristics of participants for adherence to dietary recommendation and associated factors among adult HF patients at public hospitals of Southern Ethiopia 2022 (n = 521).

Variables	Category	Frequency	Percent
Duration of diagnosis	<4years	219	42.0
	> = 4years	302	58.0
New York Heart Association functional class	I	122	23.4
	II	222	42.6
	III	151	29.0
	IV	26	5.0
History of hospitalization	Yes	210	40.3
	No	311	59.7
Number of admission n = 210	1	68	32.4
	2	85	40.5
	> = 3	57	27.1
Comorbid condition	Yes	222	42.6
	No	299	57.4
Type of chronic d/se	DM	46	8.8
	HTN	118	22.6
	HIV	5	1.0
	Asthma	10	1.9
	CKD	1	.2
	Other(epilepsy,COPD)	16	3.1
	DM+HTN	5	1.0
	HTN+CKD	23	4.4
Number of comorbid conditions	1	168	32.2
	≥2	54	10.4

### Knowledge and attitude

The study assessed participants’ understanding of heart failure and their attitudes towards recommended dietary guidelines. Using a cut-off score of 75% on the knowledge assessment, approximately 34% of participants demonstrated poor knowledge about their condition. In terms of attitudes, two-thirds (68.1%) of participants expressed unfavorable views towards the recommended diets based on the mean score of the attitude assessment.

### Dietary adherence

Adherence to the recommended low-sodium, fat-free, fluid-restricted diet with inclusion of legumes, vegetables, and fruits was assessed. Participants were categorized as "adherent" if they reported following a recommendation "always or mostly and "non-adherent" otherwise. Adherence scores ranged from 20.3% (vegetables and fruits) to 60.3% (fat-free diet), indicating generally low adherence across most dietary components. Notably, only 8.1% of participants achieved good adherence to all dietary recommendations (Table 3).

**Table 3:** Adherence status of participants for dietary components designed for adherence to dietary recommendation and associated factors among HF patients at public hospitals of Southern Ethiopia 2022 (n = 521).

Diet parameters	Adhered		Not Adhered	
	N	%	N	%
Low salt	288	55.3	233	44.7
Restricted fluid	184	35.3	337	64.7
Fat free	314	60.3	207	39.7
legumes	126	24.2	395	75.8
Vegetable and fruit	106	20.3	415	79.7
With all above parameters	42	8.1	116	22.3

**Fig 1:**
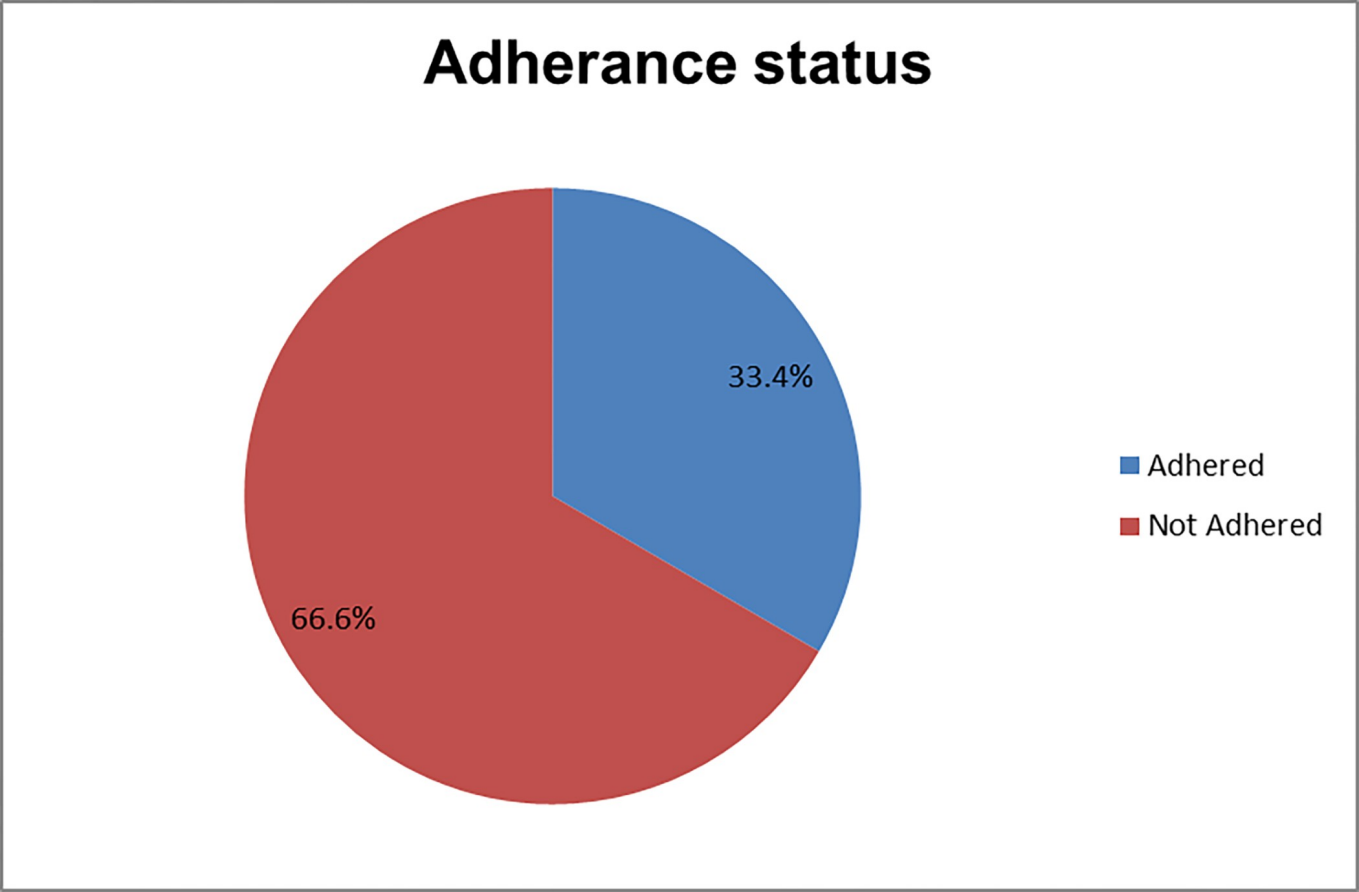
Adherence status of participants for adherence to dietary recommendation and associated factors among adult HF Patients at public hospitals of Southern Ethiopia 2022 (n = 521).

Only 33.4% (95% CI: 29–37) of respondents met their recommended diet (Fig 1).

### Factors associated with dietary adherence

Bivariate analysis explored potential factors influencing dietary adherence, including age, education, residence, occupation, hospitalization history, comorbidity, knowledge, and attitude. Multivariate logistic regression then identified factors with statistically significant associations with adherence (p-value < 0.05). These factors were age, history of hospitalization, presence of comorbidity, and attitude towards the recommended diet. Participants aged 41–60 were 1.7 times more likely to adhere compared to those aged 18–40. Furthermore, a history of hospitalization was associated with a 2.5 times greater likelihood of adherence. Interestingly, individuals with no comorbid conditions were 42% less likely to adhere than those with at least one comorbidity. Finally, a favourable attitude towards the recommended diet was a significant factor, with participants having unfavourable attitudes being 55% less likely to adhere. The detailed results of this analysis, including odds ratios and confidence intervals for each significant factor, are likely presented in Table 4

**Table 4:** Bivariable and multivariable analysis for adherence to dietary recommendation and associated factors among HF patients at public hospitals of, Southern Ethiopia 2022.

Variables		Cov-19 prevention practice				Bivariate logistic regression		Multivariate logistic regression
		Adhered		not adhered		COR	p-v	AOR(95%CI)
		N	%	N	%			
**Age**	41–60	80	39.0	125	61.0	1.532	**.041**	**1.713 (1.076 2.726)**
	>60	33	30.3	76	69.7	1.039	.881	1.464 (.800 2.679)
	18–40	61	29.5	146	70.5	1		
**Sex**	Female	82	33.2	165	66.8	.983	.927	
	Male	92	33.6	182	66.4	1		
**Marital status**	Married	123	34	239	66	1.090	.672	
	Others	51	32.1	108	67.9	1		
**Educational Status**	Able to read and write	45	31.9	96	68.1	1.042	.873	1.150 (.638 2.074)
	Elementary	42	32.8	86	67.2	1.085	.753	1.246 (.658 2.361)
	Secondary &above	42	39.3	65	60.7	1.436	**.176**	1.198 (.537 2.670)
	Unable to read and write	45	31	100	69	1		
**Residence**	Urban	102	34.5	194	65.5	1.117	.556	.863 (.530 1.405)
	Rural	72	32.0	153	68.0	1		
**Occupation**	Private employee	39	68.3	84	31.7	.500	**.038**	.495 (.227 1.079)
	Farmer and	41	32.0	87	68.0	.508	**.041**	.510 (.209 1.242)
	House wife	35	30.4	80	69.6	.471	**.027**	.466 (.194 1.123)
	Others	33	32.7	68	67.3	.523	**.060**	.633 (.249 1.607)
	Government employee	26	48.1	28	51.9	1		
Monthly Income	<median	86	33.6	170	66.4	.983	.926	
	> = Median	174	33.4	347	66.6	**1**		
Duration of diagnosis	<4years	67	30.6	152	69.4	.803	.348	
	> = 4years	107	35.4	195	64.6	1		
New York Heart association class	II	80	36	142	64	1.294	.286	
	III &IV	57	32.2	120	67.8	1.091	.731	
	I	37	30.3	85	69.7	1		
History of admission	Yes	86	41	124	59	1.76	**0.003**	**2.519 (1.574 4.031)**
	No	88	28.3	223	71.7	1		
Comorbidity	No	96	32.1	203	67.9	.873	**.046**	**.576 (.360 .924)**
	Yes	78	35.1	144	64.9			
Knowledge	Poor	47	26.6	130	73.4	.618	**.018**	.676 (.433 1.057)
	Good	127	36.9	217	63.1			
Attitude	Unfavorable	78	25.6	227	74.4	.430	**.000**	**.450 (.304 .664)**
	Favorable	96	44.4	120	55.6	**1**		

## Discussion

This study investigated dietary adherence and its associated factors among heart failure (HF) patients in Southern Ethiopia. These findings highlight the presence of multiple health concerns among some participants and concerning gaps in knowledge, unfavorable attitudes towards dietary recommendations, and overall low adherence to specific dietary components. Additionally, the study identified factors influencing adherence, including age, hospitalization history, presence of comorbidities, and attitude towards the recommended diet.

The study revealed variations in adherence across dietary components, with the lowest adherence observed for vegetables and fruits. Furthermore, only a small percentage of participants achieved good adherence to all recommendations. About thirty three percent of respondents met adherence to their recommended diet. Our findings is consistent with previous research by Marti, Georgiopoulou et al. [53]. However, adherence is lower compared to studies by van der Wal, van Veldhuisen et al., Conceição, Santos et al., Sewagegn, Fekadu et al., Fetensa, Yadecha et al. [1,41,46,54]. This difference might be due to variations in sample size. Studies by Conceição, Santos et al., Sewagegn, Fekadu et al., Fetensa, Yadecha et al. [1,46,54] had smaller sample sizes compared to our study. Another potential explanation for the variation lies in the study design and coverage area. The study by van der Wal, van Veldhuisen et al. [41] was a multicenter trial involving 17 hospitals, encompassing a much larger geographical area compared to our study.

Conversely, our adherence rate was higher than the one reported in Seid, Abdela et al. [18]. This difference is likely due to the assessment parameters employed. The previous study assessed adherence to general self-care behaviours, with diet being just one component. In contrast, our study specifically focused on adherence to dietary recommendations. Overall, the findings suggest that adherence to dietary recommendations remains a challenge.

The analysis identified several factors influencing adherence. Participants aged 41–60 were more likely to adhere compared to younger participants. This finding was supported by van der Wal, van Veldhuisen et al., Sewagegn, Fekadu et al. [41,46]. This could be due to increased awareness of health risks and a greater focus on self-care with advancing age. More over as the prevalence of HF increases with aging population the issue of HF self-care adherence among the elderly will become even more important Sewagegn, Fekadu et al. [46]. On the other hand over ages, people have observed the profound link between emotions, mood, and food choices. This influence manifests in various ways, from strong cravings to subtle subconscious cues. It can be physiological, impacting appetite, or behavioral, affecting food availability Gibson [55].

Participant clients who had a history of admission were more likely to adhere to their recommended diet compared to participants who had no history of admission. This was supported by van der Wal, van Veldhuisen et al. [41]. This association could be explained by the potential for increased education and dietary counseling during hospitalization and it is clear that increasing patients’ level of knowledge about the disease is a prerequisite to improve self-care behavior Sewagegn, Fekadu et al. [46].

Interestingly, individuals with no comorbidities were less likely to adhere than those with comorbidities. This is supported by Sewagegn, Fekadu et al. [46]. This may suggest that experiencing health complications reinforces the importance of dietary changes for some patients. And this might be also due to those participants who had additional diseases may have repeated counseling and they may fear the severity. On the other hand, the finding was contradicted by Seid, Abdela et al. [45] it might be due to the previous study seeing association to adherence of general self-care behavior while the current study to adherence of only dietary recommendation.

Finally, as expected, a favorable attitude towards the recommended diet was a significant factor associated with adherence. This finding is supported by Aridi, Walker et al. [56]. The possible explanation might be a positive outlook on a recommended diet can significantly boost adherence, diet is viewed as enjoyable and tasty, it feels more like a lifestyle change than a restrictive burden. This makes it easier to resist unhealthy temptations and stay committed. People who believe the diet will improve their health or well-being are also more motivated to follow it Karimy, Koohestani et al. [57].

## Conclusion and recommendations

The study employed rigorous methods, including validated instruments, a large sample size, and systematic sampling, to ensure generalizability. Bivariate and multivariate analyses identified factors influencing dietary adherence. Ethical standards were strictly followed to maintain research integrity. However, the cross-sectional design limits establishing causal relationships between factors and adherence. Additionally, self-reported dietary data may be influenced by recall bias, and the study’s generalizability might be limited due to the specific population and healthcare setting. Further research opportunities include longitudinal studies to track adherence changes, incorporating objective measures to strengthen assessment, and conducting multicenter studies to enhance more generalizability.

This study identified low adherence to dietary recommendations among heart failure patients in Southern Ethiopia. The analysis revealed that age, hospitalization history, presence of comorbidities, and attitude towards the recommended diet were all factors influencing adherence. These findings highlight the need for multifaceted interventions that target negative attitudes, provide support to overcome challenges associated with dietary changes, and consider the specific needs of different patient subgroups.

To improve dietary adherence in this population, strategies to address negative attitudes towards the diet, such as recipe modifications and culturally-appropriate meal planning, are crucial. Ongoing support through groups, technology, and healthcare visits can further enhance adherence. Finally, tailoring interventions for specific patient subgroups based on factors like age and comorbidities is important. This finding suggests that younger patients might require additional support or targeted interventions to overcome challenges in adhering to dietary recommendations. Additionally, while the number of participants from urban and rural areas was similar, further analysis could explore potential variations in adherence patterns between these demographics in future studies. Future research should focus on understanding the reasons behind low adherence through qualitative studies and evaluating the effectiveness of interventions on long-term outcomes. By implementing these recommendations, healthcare professionals can empower patients to better disease management, improved quality of life, and potentially reduced healthcare costs.

## Supporting information

S1 Table STROBE checklist report for the study of bridging the gap between recommendation and reality: Improving dietary adherence of heart failure populations a cross-sectional study in Ethiopia. (PDF)

S2 Table The dataset used for the study of bridging the gap between recommendation and reality: Improving dietary adherence of heart failure populations a cross-sectional study in Ethiopia. (XLS)

## Abbreviations

AHA: American Heart Association
AOR: Adjusted Odds Ratio
HF: Heart Failure
Na: Sodium
NYHA: New York Heart Association
SPSS: Statistical Package for Social Science
SSBs: Sugar-Sweetened Beverages
US: United States
